# Striatal Glutamate Release in l-DOPA-Induced Dyskinetic Animals

**DOI:** 10.1371/journal.pone.0055706

**Published:** 2013-02-04

**Authors:** Nina Nevalainen, Martin Lundblad, Greg A. Gerhardt, Ingrid Strömberg

**Affiliations:** 1 Integrative Medical Biology, Umeå University, Umeå, Sweden; 2 Basal Ganglia Pathophysiology Unit, Neuroscience Section, Department of Experimental Medical Science, Lund University, Lund, Sweden; 3 Anatomy, Neurobiology, and Neurology, University of Kentucky Medical Center, Lexington, Kentucky, United States of America; Karolinska Inst, Sweden

## Abstract

l-DOPA-induced dyskinesia is a common side effect developed after chronic treatment with 3,4-dihydroxyphenyl-l-alanine (l-DOPA) in Parkinson's disease. The biological mechanisms behind this side effect are not fully comprehended although involvement of dopaminergic, serotonergic, and glutamatergic systems has been suggested. The present study utilizes *in vivo* amperometry to investigate the impact from unilateral 6-hydroxydopamine lesions and l-DOPA (4 mg/kg, including benserazide 15 mg/kg) -induced dyskinetic behavior on striatal basal extracellular glutamate concentration and potassium-evoked glutamate release in urethane-anesthetized rats. Recordings were performed before and after local l-DOPA application in the striatum. In addition, effects from the 5-HT_1A_ receptor agonist (2R)-(+)-8-hydroxy-2-(di-n-propylamino)tetralin hydrobromide (8-OHDPAT; 1 mg/kg) was assessed on glutamate release and on dyskinetic behavior. The results revealed a bilateral ∼30% reduction of basal extracellular glutamate concentration and attenuated potassium-evoked glutamate release after a unilateral dopamine-depletion in l-DOPA naïve animals. In dyskinetic subjects, basal glutamate concentration was comparable to normal controls, although potassium-evoked glutamate release was reduced to similar levels as in drug naïve dopamine-lesioned animals. Furthermore, acute striatal l-DOPA administration attenuated glutamate release in all groups, except in the dopamine-lesioned striatum of dyskinetic animals. Co-administration of 8-OHDPAT and l-DOPA decreased dyskinesia in dopamine-lesioned animals, but did not affect potassium-evoked glutamate release, which was seen in normal animals. These findings indicate altered glutamate transmission upon dopamine-depletion and dyskinesia.

## Introduction

Patients with Parkinson's disease are treated with 3,4-dihydroxyphenyl-l-alanine (l-DOPA) in order to improve motor function. Although providing satisfactory relief of motor symptoms at early stages of the treatment, side effects referred to as l-DOPA-induced dyskinesia (LID) are seen in the majority of patients after several years of chronic l-DOPA treatment. LID manifests as uncontrollable, involuntary movements and affects up to 40% of the patients after 4–6 years and as many as 90% of the patients after 9 years of treatment with l-DOPA [1]. The biological mechanisms responsible for the upcoming of LID are not fully understood to this date, but the involvement of several distinct neurotransmitter systems has been established [2]. Apart from the dopaminergic, the serotonergic (5-HT; 5-hydroxytryptamine) and glutamatergic system have been pointed out as two likely key-players.

The 5-HT system, with origin in the raphe nuclei, has been suggested as the neuronal structure responsible for the upcoming of LID, mainly since these neurons have the ability to convert l-DOPA to dopamine as well as store and release dopamine [3], [4], [5], [6], [7], [8], [9]. It has further been proposed that dopamine release from 5-HT nerve terminals in the dopamine-lesioned striatum would give rise to fluctuating striatal dopamine levels and thus result in pulsatile postsynaptic receptor stimulation and the development of LID. In line with this hypothesis, it has been demonstrated that lesion of the 5-HT system or decreased 5-HT neuron activity via autoreceptor activation attenuates dyskinetic behavior in animal models of LID [10], [11]. The mechanisms referred to is that the dopamine levels are reduced and thereby LID is counteracted, indicating that the dopamine levels are abnormally high in the striatum of dyskinetic animals. However, the release of dopamine after acute l-DOPA in dyskinetic animals is reduced, which does not support this theory [12], [13]. Furthermore, decreased 5-HT activity seems to be one of the pathological features as it has been documented that in addition to the dopaminergic degeneration, the 5-HT system degenerates in parkinsonian patients [14], [15], [16]. In animal experiments, chronic l-DOPA treatment decreases 5-HT and 5-HT metabolite levels in several brain areas [17]. These observations raise the question whether it is the dopamine derived from 5-HT neurons that gives rise to LID, or if it might be dysmodulation of the 5-HT system.

Apart from the influence of the 5-HT system, increased corticostriatal glutamate neurotransmission and receptor activation and function has been strongly associated with the upcoming and maintenance of LID, as shown both in animal models and patients [18], [19], [20], [21], [22], [23]. It has been postulated that increased corticostriatal signaling might contribute to abnormal motor performance by causing an imbalance between the striatal output pathways, and indeed, the overactivity of the dopamine D_1_-receptor-mediated direct pathway has been correlated to expression of LID [24], [25], [26], [27], [28]. In further support, several investigations have provided evidence on relieved LID upon antagonism on glutamate receptors [29], [30], [31], [32], [33], [34], [35], [36], and in fact, the only antidyskinetic drug currently used in patients is the glutamate *N*-methyl-D-aspartate (NMDA) receptor antagonist amantadine [37], [38]. However, dysregulation of the 5-HT system may also affect the glutamate levels as the cortical neurons express 5-HT_1A_ receptors [39], and a 5-HT receptor agonist inhibits striatal glutamate release [40], [41], [42]. Therefore, decreased 5-HT nerve terminal density in Parkinson's disease may affect the striatal glutamate influx. Thus, both the 5-HT and glutamatergic system seems to be critically involved in the pathogenesis of LID, however, the mechanisms for interaction between these circuits are still elusive. The aims of the present study were to further investigate the effects from dopamine depletion, chronic l-DOPA treatment, and 5-HT_1A_ receptor stimulation on striatal glutamate levels. For these purposes, *in vivo* amperometry was employed for subsecond quantification of basal glutamate concentration and glutamate release in the striatum of urethane-anesthetized drug naïve hemiparkinsonian and chronically l-DOPA-treated dyskinetic rats. Data were collected before and after acute striatal l-DOPA infusion and the 5-HT_1A_ receptor agonist (2R)-(+)-8-hydroxy-2-(di-n-propylamino)tetralin hydrobromide (8-OHDPAT) was co-administered to evaluate the impact of 5-HT_1A_ receptor activation during l-DOPA loading on striatal glutamate concentration. Furthermore, a behavioral study was carried out to evaluate effects of 8-OHDPAT on LID.

## Materials and Methods

### Animals

**Figure 1:**
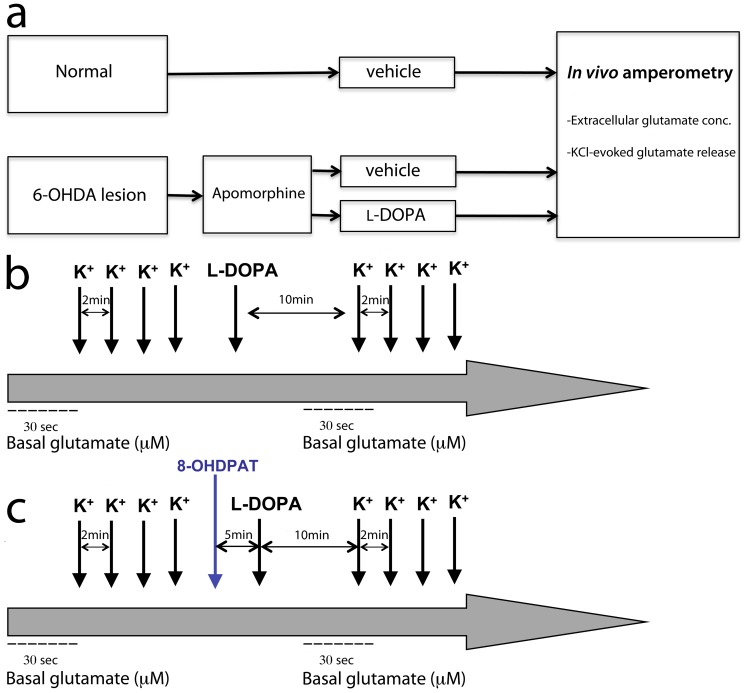
Illustration of the study design (a) and experimental setup during *in vivo* recordings of basal glutamate concentration and glutamate release stimulated by KCl-ejections (K+) before and after striatal l-DOPA application (b) and the administration of 8-OHDPAT (c).

Female Sprague-Dawley rats (Taconic Farms Inc.; Denmark) were used in this study. Animals were treated and housed in accordance with internationally accepted guidelines and all experiments had been approved by the local ethics committee, Umeå Ethics Committee for Animal Studies (permit number: A23-08). Food and water was provided *ad libitum* and animals were housed under 12∶12 h light-dark conditions. For the experimental setup, animals of similar age were divided into three separate treatment groups that constitute of unilateral 6-OHDA-lesioned animals that received either chronic l-DOPA (n = 14) or vehicle (0.9% NaCl; n = 5) injections, and normal rats receiving vehicle (n = 6), see Fig. 1a.

### Chemicals

Drugs and chemicals used throughout this study were purchased from Sigma (Stockholm, Sweden), except the enzyme l-glutamate oxidase (Seikagaku, US Biologicals, Massachusetts, USA) and the 5-HT_1A_ agonist 8-OHDPAT (TOCRIS Bioscience, BioNuclear Scandinavia AB, Stockholm, Sweden). All physiologically (*in vivo*) applied solutions were dissolved in 0.9% NaCl (pH = 7.4), whereas the standard solutions for calibration and coatings i.e. solutions for *in vitro* use were dissolved in distilled deionized water.

### 6-OHDA injection and apomorphine-induced rotational behavior

Dopamine depletions were performed according to the 6-hydroxydopamine (6-OHDA) rat model [43]. When performing the dopamine lesion, animals (∼150 grams) were anesthetized with 4% isoflurane and placed in a stereotaxic frame. An incision was made in the scalp and a hole was drilled in the skull at 4.4 mm posterior and 1.2 mm lateral from the bregma position. A Hamilton needle was lowered −7.8 mm below the dura mater and 8 µg of 6-OHDA (dissolved in 4 µl saline containing 0.02% ascorbic acid) was injected into the medial forebrain bundle at a rate of 1 µl/min. The needle was left in place for 3 min before being withdrawn.

Two weeks after the intracerebral 6-OHDA-injections, rats were evaluated for apomorphine-induced rotational behavior in order to determine lesion severity. Rats were placed in separate bowls and left to acclimatize for approximately 15 min before receiving a subcutaneous injection of 0.05 mg apomorphine/kg bodyweight. Total contralateral turns to the lesioned hemisphere were counted, and animals that made >450 turns during 70 min were included in this study. It has previously been demonstrated that this value corresponds to at least 90% striatal dopamine-depletion [44].

### l-DOPA treatment and dyskinesia ratings

Rats with severe dopamine lesions received chronic l-DOPA treatment (4 mg/kg) plus benserazide-hydrochloride (15 mg/kg) or vehicle beginning at 1.5 months after the dopamine depletion. The animals were given l-DOPA or vehicle once daily for 14 days and were evaluated for the expression of abnormal involuntary movements (AIMs) on days 1, 4, 7, 10, and 14 according to previously described protocols [45]. Briefly, rats were injected subcutaneously (2 ml/kg bodyweight) with l-DOPA or vehicle and placed in separate cages. The animals were evaluated for the expression of axial, orolingual, and forelimb AIMs for 1 min every 20th min during 3 h, starting 20 min after the injection. Scores from 0–4 were given for each subtype based on its severity and duration (0 = not present, 1 = present during less than half of the observation time, 2 = present during more than half of observation time, 3 = continuous, but interrupted by external distraction, 4 = continuous, not interrupted by external distraction), thus the total maximum score per animal during one session is 108. Animals were considered dyskinetic when displaying scores of ≥2 on at least two of the three subtypes during one observation period. Following 14 days of daily treatment, rats received l-DOPA 2–3 times per week and the AIMs ratings were carried out every 10^th^ day to ensure stable status of dyskinesia. The dyskinetic animals included in the amperometric and behavioral studies expressed total AIMs scores of 38.71±6.89 and 41.67±5.53, respectively, at their last testing session. Animals received their last l-DOPA injection 2 days before electrochemical recordings. Recordings were performed between 4–8 months after the initiation of l-DOPA/vehicle treatment, and all animals were age-matched. For the behavioral study, 8-OHDPAT (1 mg/kg) was subcutaneously administered 5 min prior to l-DOPA. Behavioral assessments with 8-OHDPAT were performed between 8 months after the initiation of l-DOPA/vehicle treatment.

### *In vivo* amperometry

#### Preparation and calibration of electrodes

A detailed description of the entire methodological process of *in vivo* amperometric recordings of glutamate (preparation of electrodes, calibration, and recordings *in vivo* etc.) has been published previously and was followed with some minor modifications [46]. *In vivo* amperometry was conducted with enzyme-coated ceramic-based microelectrode arrays [47], [48] to monitor basal glutamate concentration and glutamate release in anesthetized animals. A self-reference recording technique was employed to selectively measure glutamate by removing background noise and non-specific signals from interfering molecules [49]. Electrodes (Thin-Film Technologies, Inc., Buellton, CA, USA) with four recording sites (333×15 µm) arranged as two pairs (sites separated by 30 µm for each pair) were prepared so that one pair of recording sites were able to detect glutamate whilst the other pair of channels, located 100 µm from the first pair, acted as sentinel sites. The glutamate recording sites were coated with a solution of l-glutamate oxidase (1%), bovine serum albumin (BSA, 1%), and glutaraldehyde (0.125%). Hence, when glutamate reaches the electrode surface, it is degraded by the l-glutamate oxidase into α-ketoglutarate and hydrogen peroxide (H_2_O_2_), where the latter molecule is oxidized and the electrons freed in this process is detected by the electrode [50]. The sentinel sites were coated with a BSA/glutaraldehyde solution, thus resulting in a protein layer with similar properties as at the recording sites but lacking the ability to record glutamate. Consequently, the current recorded by the sentinel sites subtracted from the current recorded by the glutamate detecting sites results in a signal specific for glutamate. After the coating procedure, electrodes were let to dry for at least 72 h in a dry and clean atmosphere. An additional coating procedure was applied where all four recording sites were electroplated with 1,3-phenylenediamine (m-PD, 5 mM) to block interfering molecules, such as ascorbic acid, from reaching the recording sites by size-exclusion. The electrodes were utilized for amperometric recordings earliest 24 h after the electroplating procedure.

Amperometric recordings were preceded by a calibration procedure where the electrode properties were challenged to ensure selective detection of glutamate. Electrodes were calibrated in 40 ml phosphate buffered saline (PBS, 0.05 M, pH = 7.4) at 37°C against an Ag/AgCl reference electrode. A constant potential of 0.7 V was applied at 2 Hz using the Fast Analytical Sensing Technology (FAST-16) system and software (Quanteon, LLC, Nicholasville, KY, USA) and the signal was amplified 500× (2 nA/V) via the headstage. Standard solutions of ascorbic acid and glutamic acid were added to the beaker to produce a final concentration of 250 µM of ascorbic acid and 60 µM of glutamic acid, where the latter was achieved by three subsequent 20 µM increments (Fig. S1). The resulting current produced upon each addition was measured and electrodes were included when detecting glutamate in a linear manner (R^2^>0.99) with selectivities of >20∶1 over ascorbic acid, and with a limit of detection <2 µM with a signal-to-noise level set at 3∶1. The electrodes utilized in the present study expressed selectivities of 66.905±9.112 over ascorbic acid, limit of detection of 0.774±0.110 and R^2^ 0.998±0.001. Standard solutions of dopamine and H_2_O_2_ were added to the beaker (2 and 8.8 µM, respectively) at the end of the calibration, to assure impermeability of larger molecules and to test electrode sensitivity to the reporter molecule H_2_O_2_.

#### *In vivo* recordings

Two micropipettes with tip diameters of 10–15 µm were filled with potassium chloride (KCl, 70 mM) and l-DOPA (2.5 mM) respectively, and mounted parallel to each other with sticky wax (Kerr Lab Corporation, Orange, CA, USA). The micropipettes were then mounted at a distance of 50–100 µm from the electrode surface in a centered position between the four recording sites. The tip of the electrode/micropipette-assembly was placed in PBS until used for *in vivo* recordings.

Animals were anesthetized with urethane (1.25–1.5 g/kg, i.p.). When under surgical anesthesia, animals were tracheotomized to facilitate spontaneous breathing and placed in a stereotaxic frame. The scalp was removed and two holes were made with a dental drill in the bone overlying the striatum bilaterally. An additional burr hole was made caudally, at a position remote from the recording sites, for insertion of a miniature Ag/AgCl electrode. The dura mater was removed before lowering the electrode into the striatum with a microdrive. Recordings were performed at four striatal positions in each hemisphere (ML: ±2.6 mm, AP: ±0/+1 mm, DV: −3.5 and −4.5 mm, calculated from the bregma). At each striatal recording site, a stable baseline was acquired, and then basal glutamate concentration and glutamate release was recorded before and after local infusion of l-DOPA, thus, monitoring effects on glutamate levels during OFF and ON l-DOPA. When recording glutamate release, calibrated volumes of KCl (100 nl) and l-DOPA (100 nl) was pressure-ejected at the recording sites. KCl was ejected 4–5 times before and after a l-DOPA ejection with 2-min intervals. Upon l-DOPA ejection, 10 min was let to pass before re-stimulating with KCl (Fig. 1b). It has been demonstrated that l-DOPA can be taken up and converted to dopamine during a 10-min period, and then being released by KCl [13]. As a final challenge, in normal animals and in the dopamine-lesioned striata of l-DOPA naïve and dyskinetic animals, l-DOPA loading was preceded (5 min) by a subcutaneous injection of the 5HT_1A_ receptor agonist 8-OHDPAT (1 mg/kg; Fig. 1c). 8-OHDPAT was applied systemically to achieve an overall effect by the 5-HT_1A_ receptor agonist, while l-DOPA was applied locally to monitor the glutamate levels in l-DOPA-free and on-drug at each recording site. The hemisphere and striatal position for the first measurement was alternated between the subjects of each experimental group. The placement of the electrodes was confirmed to be in the right position after the recordings.

### Statistical analysis

Processing of the raw data from recordings and calculations were performed with the F.A.S.T Analysis software. In order to determine basal extracellular glutamate concentration, the baseline value was averaged over 30 sec before and after l-DOPA infusion. This was performed by subtracting the current recorded by the sentinel channel from the signal taken up by the recording channel, hence providing assessment of the resting extracellular glutamate concentration (µM). Upon each KCl-stimulation, an increase in extracellular glutamate concentration was observed, and the maximum glutamate concentration reached (calculated from the baseline) was defined as the maximum peak amplitude and referred to as glutamate release (µM).

Data were analyzed with one- or two-factor analysis of variance (ANOVA), Student's t-test, and paired t-test. Two-factor ANOVA followed by Bonferroni post hoc test was performed to assess the effects of the different treatment strategies between groups and the effects of acute l-DOPA and 8-OHDPAT administrations on glutamate levels. One-way ANOVA followed by Bonferroni post hoc test, or Student's t-test was carried out to assess individual differences between and within groups, respectively. A paired t-test was performed to assess the effects of 8-OHDPAT on LID. All values are presented as mean values ± standard error of mean, and the significance level was set at p<0.05.

## Results

### Effects on basal extracellular glutamate concentration

**Figure 2:**
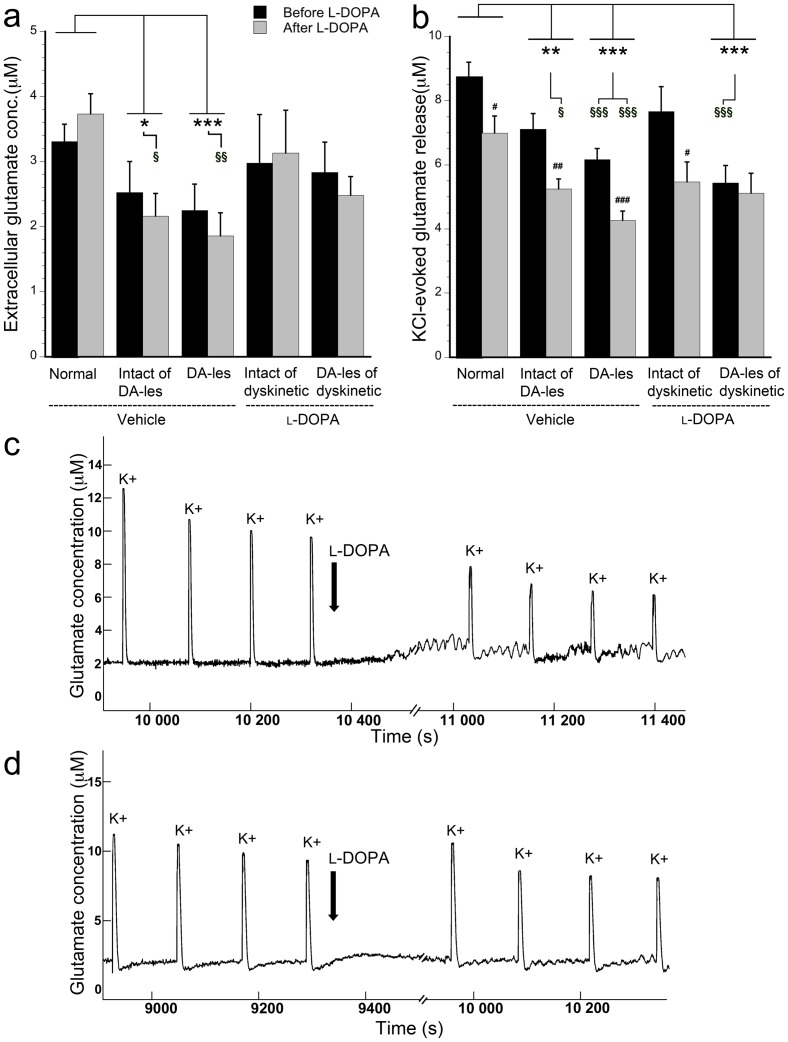
Quantification (in µM) of basal extracellular glutamate concentration (a) and KCl-evoked glutamate release (b) before and after acute l-DOPA administration in normal rats and in bilateral striata of vehicle- and l-DOPA-treated animals. Extracellular glutamate concentration was estimated by averaging the baseline and glutamate release was observed upon KCl-ejections (K+) during *in vivo* recordings, here in normal striatum (c) and dopamine-lesioned striatum of a dyskinetic subject (d). #p<0.05, ## p<0.01, and ###p<0.001 compared to before acute l-DOPA administration in the same group of animals. * p<0.05, ** p<0.01, and *** p<0.001 for comparisons between groups with two-factor ANOVAs and § p<0.05, §§ p<0.01, and §§§ p<0.001 for comparisons between groups with one-way ANOVA. Dopamine-lesioned striata is abbreviated DA-les.

In the striatum of normal vehicle-injected animals, the basal extracellular glutamate concentration was measured to approx. 3–4 µM before any drugs were applied (Fig. 2a). In the unilaterally dopamine-depleted vehicle-treated animals, the glutamate levels were decreased approx. 30% when compared to normal animals. This effect was seen bilaterally i.e. both in the dopamine-depleted (F = 5.442, p<0.001, two-factor ANOVA) and intact (p = 0.045) striata and was most prominent after acute l-DOPA application (F = 5.410, p = 0.026 in intact and p = 0.001 in lesioned striatum, one-way ANOVA; Fig. 2a). Chronically l-DOPA-treated dyskinetic animals displayed basal extracellular glutamate concentration that was comparable to those observed in normal animals (Fig. 2a). It should also be noted that the acute l-DOPA administration at the striatal recording site did not give rise to changes in the basal glutamate concentration when comparing with levels before l-DOPA loading in any of the groups examined (p = 0.699). In summary, these findings demonstrate that lesioning of the nigrostriatal dopamine system attenuates basal glutamate concentration, not only in the dopamine-depleted striatum but also in the contralateral hemisphere that still possesses an intact dopamine innervation, when comparing with normal animals. Upon chronic l-DOPA treatment of dopamine-lesioned animals, on the other hand, the striatal glutamate concentration was similar to levels recorded in normal animals.

### Effects of l-DOPA on KCl-evoked glutamate release

Striatal KCl ejections (100 nl, 70 mM) gave rise to glutamate release of varying amplitudes when comparing recordings performed in different groups (F = 13.836, p<0.001, two-factor ANOVA). In the unilaterally dopamine-depleted vehicle-treated animals, significantly reduced amplitude of glutamate was recorded bilaterally upon KCl ejections compared to normal striatum (intact striatum p = 0.001 and lesioned striatum p<0.001; Fig. 2b). More specifically, the attenuating effects were found before acute l-DOPA administration in the dopamine-lesioned striatum (F = 8.399, p<0.001, one-way ANOVA) and bilaterally after the striatal l-DOPA loading (F = 6.361, p = 0.044 for intact and p<0.001 for lesioned striatum, one-way ANOVA). A similar observation was made in the dopamine-lesioned striatum of chronically l-DOPA-treated dyskinetic animals, where the peak amplitude for glutamate release was significantly reduced compared to normal animals (F = 13.836, p<0.001, two-factor ANOVA; Fig. 2b). The reduction in glutamate release was found before l-DOPA administration (F = 8.399, p<0.001, one-way ANOVA).

The acute striatal application of l-DOPA altered the peak amplitude of KCl-evoked released glutamate (F = 16.114, p<0.001, two-factor ANOVA), such that an attenuating effect on glutamate release was observed in the striatum of normal animals (t(198) = 2.569, p = 0.011; Fig. 2b,c) as well as in both striata of unilaterally dopamine-depleted vehicle-treated animals (t(106) = 3.256, p = 0.002 in intact and t(198) = 4.262, p<0.001 in lesioned). A similar reduction was observed in the intact striatum (t(25) = 2.233, p = 0.035), but not in the dopamine-lesioned striatum of dyskinetic animals (t(92) = 0.387, p = 0.700; Fig. 2b, d). To exclude that the diminishing effect of acute l-DOPA administration on KCl-evoked glutamate release was not an artifact produced by the ejected volume *per se*, local ejections with vehicle (100 nl) was performed in the striatum of normal animals. The results revealed that locally applied vehicle did not affect the KCl-evoked glutamate release (t(18) = 0.104, p = 0.919).

### Effects of a 5-HT_1A_ receptor agonist on KCl-evoked glutamate release and dyskinesia

Administration of 8-OHDPAT did not affect the basal extracellular glutamate concentration in any of the treatments, hence, the recorded glutamate levels were similar (p = 0.931) after the administration of 8-OHDPAT in combination with l-DOPA as seen before drugs or after acute l-DOPA administration.

**Figure 3:**
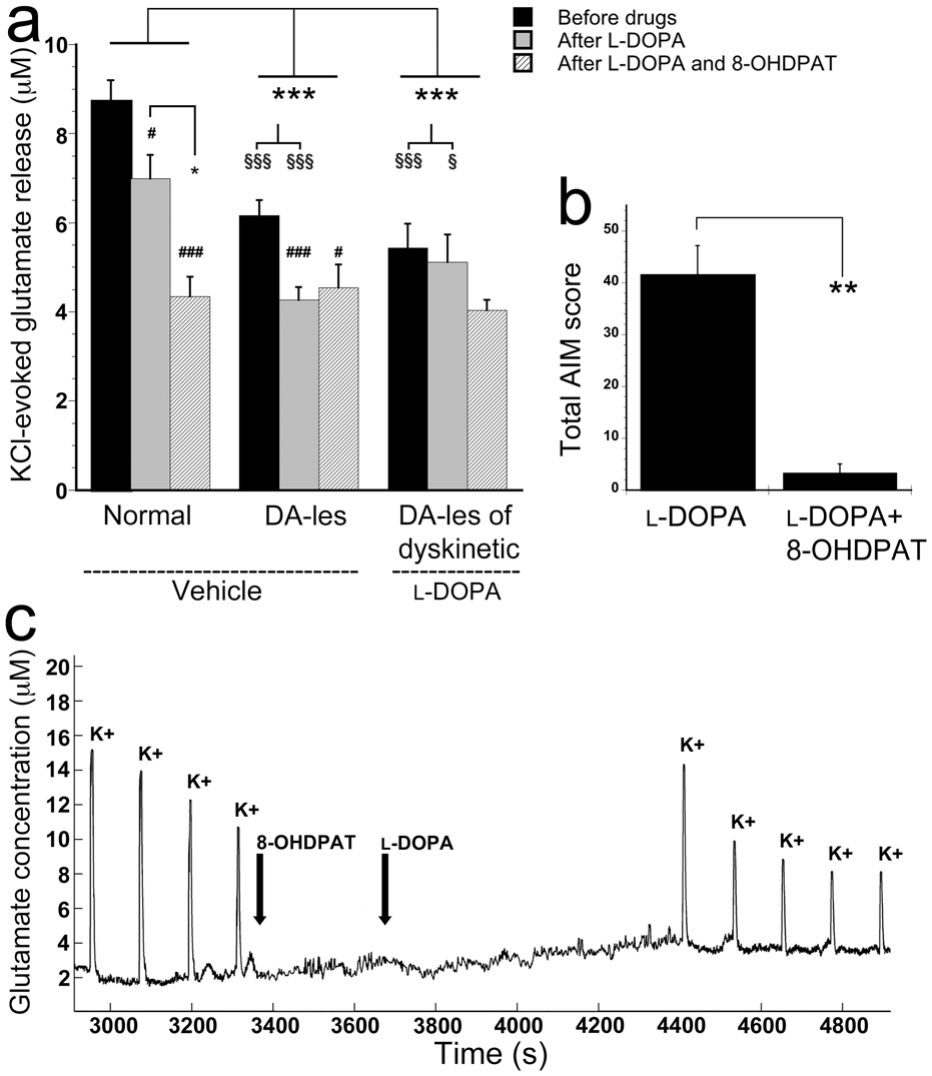
The effects of systemically administered 8-OHDPAT on KCl-evoked (K+) glutamate release (µM) during striatal l-DOPA-loading in normal and dopamine-lesioned striata of vehicle- and l-DOPA-injected animals (a) and on AIM scores in dyskinetic animals (b). 8-OHDPAT, systemically administered 5 min prior to striatal l-DOPA ejection, further reduced glutamate release seen after local l-DOPA application in normal (a, c), but not in dyskinetic animals, even though dyskinetic behavior was attenuated (b) when applying the drugs systemically. # p<0.05 and ### p<0.001 compared to before acute l-DOPA administration in the same group of animals. * p<0.05 and *** p<0.001 for comparisons between groups with 2-factor ANOVA, within groups with one-way ANOVA, and paired t-test. § p<0.05, §§§ p<0.001 for comparisons between groups with one-way ANOVA. Dopamine-lesioned striata is abbreviated DA-les.

To study the impact of 5-HT_1A_ receptor activation during local l-DOPA loading on KCl-evoked glutamate release, 8-OHDPAT (1 mg/kg) was systemically administered 5 min prior to striatal recordings were initiated (Figs. 1c and 3c). These trials were performed in the dopamine-lesioned striata of vehicle- and chronically l-DOPA-treated animals, respectively, and compared with the effects seen in normal striatum (Fig. 3a). In the normal striatum, administration of 8-OHDPAT and l-DOPA significantly decreased glutamate release when compared to levels detected before application of drugs (F = 10.634, p<0.001, one-way ANOVA; Fig. 3a,c) and decreased glutamate release after local l-DOPA administration (p = 0.029; Fig. 3a). Hence, the attenuating effect of l-DOPA on glutamate release was further potentiated by 8-OHDPAT in normal striatum. In the dopamine-lesioned striata of both vehicle-treated and chronically l-DOPA-treated animals, 8-OHDPAT did not alter the KCl-evoked glutamate release compared to what was monitored after l-DOPA alone (Fig. 3a).

During behavioral evaluation, the antidyskinetic effect of 8-OHDPAT was demonstrated. Thus, injection of 8-OHDPAT (1 mg/kg, s.c.) 5 min prior to l-DOPA (4 mg/kg, s.c.) significantly reduced the AIMs scores (t(5) = 6.109, p = 0.002, paired t-test; Fig. 3b).

## Discussion

In this study, striatal basal glutamate concentration and release were quantified utilizing *in vivo* amperometry with focus on effects from a unilateral dopamine lesion and subsequent l-DOPA treatment as well as the impact from 5-HT_1A_ receptor activation. The basal striatal extracellular glutamate concentration was bilaterally attenuated in unilaterally dopamine-depleted animals, whilst upon chronic l-DOPA treatment, similar glutamate levels were observed as in normal controls. KCl-evoked glutamate release was significantly decreased both in drug naïve and dyskinetic dopamine-lesioned animals when compared to normal subjects. Moreover, acute administration of l-DOPA reduced striatal glutamate release in normal and dopamine-lesioned drug naïve animals, while no effect was found in the dopamine-lesioned striatum of dyskinetic animals. Co-administration of 8-OHDPAT and l-DOPA further potentiated the decrease in normal animals but not in dopamine-lesioned striatum, neither in dyskinetic nor in l-DOPA naïve animals.

Basal extracellular glutamate concentration was bilaterally attenuated in unilaterally dopamine-depleted drug naïve animals when compared to normal controls. Previous investigations have demonstrated contradictory results in the matter of striatal glutamate activity upon a dopamine lesion, where increased [51], [52], decreased [53], and no effects [18], [54], [55], [56] on glutamate levels have been reported. These discrepancies might be a consequence of the time point chosen for recordings following the 6-OHDA lesion, as basal glutamate levels are decreased at later stages post-dopamine lesions compared to earlier time points [57]. In the present study, all recordings were performed 4–8 months post-lesion and thus, the reduced basal glutamate levels might be a consequence of the later time point chosen for recordings. Interestingly, the basal glutamate levels were neither changed in dyskinetic animals nor were these affected by acute l-DOPA application, although it has previously been reported that chronic l-DOPA treatment increases the basal glutamate levels which is seen during dyskinetic behavior [18], [58]. The opposing results might be explained by differences in methodologies used. In microdialysis studies, the sampling time is up to 20 min while in the present study, the KCl-evoked glutamate release and reuptake occurred within 10 sec. Furthermore, the different results in released glutamate may also reflect the different procedures for application of acute l-DOPA, i.e. local application as in the present study versus systemic injection [58]. Accordingly, the local application might not give rise to the high levels accumulated after systemic injection. However, the amount of l-DOPA applied locally demonstrated effects on peak amplitude of glutamate in l-DOPA naïve animals, thereby implicating that the amount of l-DOPA that was injected was sufficient to achieve an effect.

The bilateral effects on basal extracellular glutamate concentration in the dopamine-depleted animals were unanticipated since the animals received unilateral dopamine lesions. Accordingly, it seems as the extracellular glutamate levels are regulated to gain bilateral homeostasis, even though there are morphological differences between the hemispheres. This could, with great likelihood, occur when considering the fact that aside from the ipsilateral corticostriatal projections, a significant proportion of the corticostriatal afferents have their origin in the contralateral hemisphere [59], [60], [61], [62]. Additional interhemispheric connections are probably involved, as electrophysiological recordings have demonstrated bilateral effects on neuronal activity of several basal ganglia nuclei upon unilateral dopamine depletion [63]. In addition, bilateral effects on glutamate levels in unilaterally dopamine-depleted animals have indeed been demonstrated before [52].

Administration of the NMDA receptor antagonist amantadine hampers dyskinetic behavior [34], [38], [55], [64], [65]. Involvement of glutamate in LID is further demonstrated using the more specific NMDA receptor antagonist MK801 [66] as well as the metabotropic glutamate receptor 5 (mGluR5) antagonist [29], [35], [67], which also attenuates LID. Accordingly, blocking the glutamate receptors ameliorates dyskinesia, at least partly, indicating an antidyskinetic effect from regulation of the glutamate release. In the present study, acute application of l-DOPA reduced the amplitude of glutamate release in all striata, except in the dopamine-lesioned hemisphere of dyskinetic animals. The reason for the lack of effect in dyskinetic animals is still elusive, however, amantadine also enhances the dopamine levels in the dopamine-depleted striatum after l-DOPA injection [68]. The striatal glutamate nerve terminals express dopamine D_2_ receptors and activation of these receptors is believed to inhibit corticostriatal glutamate release [42]. Therefore the absence of effects of l-DOPA, found in the present study, on glutamate release in the dopamine-depleted striatum of dyskinetic animals might be an indication of poor l-DOPA conversion, which has been demonstrated previously [12], [13], [17]. In addition, the activity of the enzyme that converts l-DOPA to dopamine is reduced in dyskinetic animals [69] and further strengthens this theory. Thus, the lack of reduction in glutamate release found in dyskinetic animals after l-DOPA loading support the behavioral studies where glutamate receptor antagonists may reduce dyskinetic behavior.

To evaluate the mechanisms by which the 5-HT_1A_ receptor agonist can reduce LID, as demonstrated here and by others, the dose of 1 mg/kg 8-OHDPAT was utilized. This concentration was chosen since doses of ≥0.2 mg/kg give rise to behavioral effects in rats and marmosets referred to as serotonergic syndrome [10], [70], which is known to arise due to cortical 5-HT_1A_ receptor stimulation [39], [71], [72]. It has been demonstrated that the 5-HT_1A_ agonist inhibits striatal glutamate release and that direct infusion of 8-OHDPAT into the primary motor cortex attenuates LID [40], [41], [73]. One study illustrated both events, where systemic administration of 8-OHDPAT at the same dose as used in the present study not only attenuated LID but also the striatal glutamate levels [58]. However, the action of the 5-HT_1A_ receptor agonist to counteract LID has also been referred to reduced dopamine levels by inhibiting l-DOPA-generated dopamine release from 5-HT nerve terminals [10], [74], [75]. In line with these observations, the results presented here revealed that 8-OHDPAT further attenuated the reduction in KCl-evoked glutamate release, which was seen after l-DOPA application in normal animals, while no effects were found in the dopamine-lesioned striata of neither l-DOPA naïve nor dyskinetic rats. Thus, even though the 5-HT_1A_ receptor agonist may affect the glutamate release by direct effects on the corticostriatal neurons, this was not the case in dopamine-depleted striatum. In normal striatum, dopamine can be released from intact dopamine nerve fibers and thereby reduce glutamate release via the D_2_ receptors on the corticostriatal glutamate fibers, while dopamine release generated from exogenous l-DOPA in the dopamine-depleted striatum is inhibited by the 5-HT_1A_ receptor agonist. It has been reported that reduction of LID can occur via synergistic actions of a NDMA antagonist and 8-OHDPAT [76]. The administration of 8-OHDPAT did not affect glutamate release in the present study, which could be assigned that the local administration of l-DOPA might give insufficient doses, however, it is not likely since it was possible to regulate glutamate release in normal animals. While this serves explanatory for the effects of 5-HT_1A_ receptor agonist on glutamate release, it is still elusive whether it is dopamine or glutamate that is the most important neurotransmittor to be regulated in LID. Most likely it is a combination since dopamine D_1_ and NMDA receptor complexes are downregulated in dyskinesia [77].

In conclusion, the present study demonstrates that glutamate release is reduced in the dopamine-depleted striatum, and that l-DOPA application can further attenuate the glutamate release in dopamine-lesioned striatum of drug naïve but not in dyskinetic animals. These data indicate that glutamate levels are not different in dopamine-lesioned drug naïve and dyskinetic animals, although there is a loss of regulation of glutmate levels upon l-DOPA administration in dyskinetic animals. This work thereby support behavioral studies where glutamate receptor antagonists may reduce dyskinetic behavior by blocking the input on postsynaptic glutamate receptors on the striatal output neurons, as the critical mechanism to counteract LID might be regulation of glutamate levels. Furthermore, 8-OHDPAT did not affect the glutamate release in dopamine-depleted striatum, suggesting that the effects of 8-OHDPAT when reducing dyskinetic behavior is not a consequence from regulation of the glutamate input to the striatum.

## Supporting Information

Figure S1 **Calibration of microelectrode arrays.** The electrodes were coated with glutamate oxidase and 1,3-phenylenediamine, resulting in selective recordings of glutamate (Glu, 20 µM per addition) by blocking larger molecules, such as ascorbic acid (AA, 250 µM) and dopamine (DA, 2 µm) from the electrode surface. The reporter molecule for the degradation of glutamate by glutamate oxidase, hydrogen peroxide (H_2_O_2_, 8.8 µM), was added to the calibration beaker to assure detection by the electrode.
(TIF)
