# Supplementary figures and images for: Striatal Glutamate Release in l-DOPA-Induced Dyskinetic Animals
Source: PLoS One. 2013 Feb 4;8(2):e55706. doi: 10.1371/journal.pone.0055706 (PMC3563586; doi:10.1371/journal.pone.0055706)

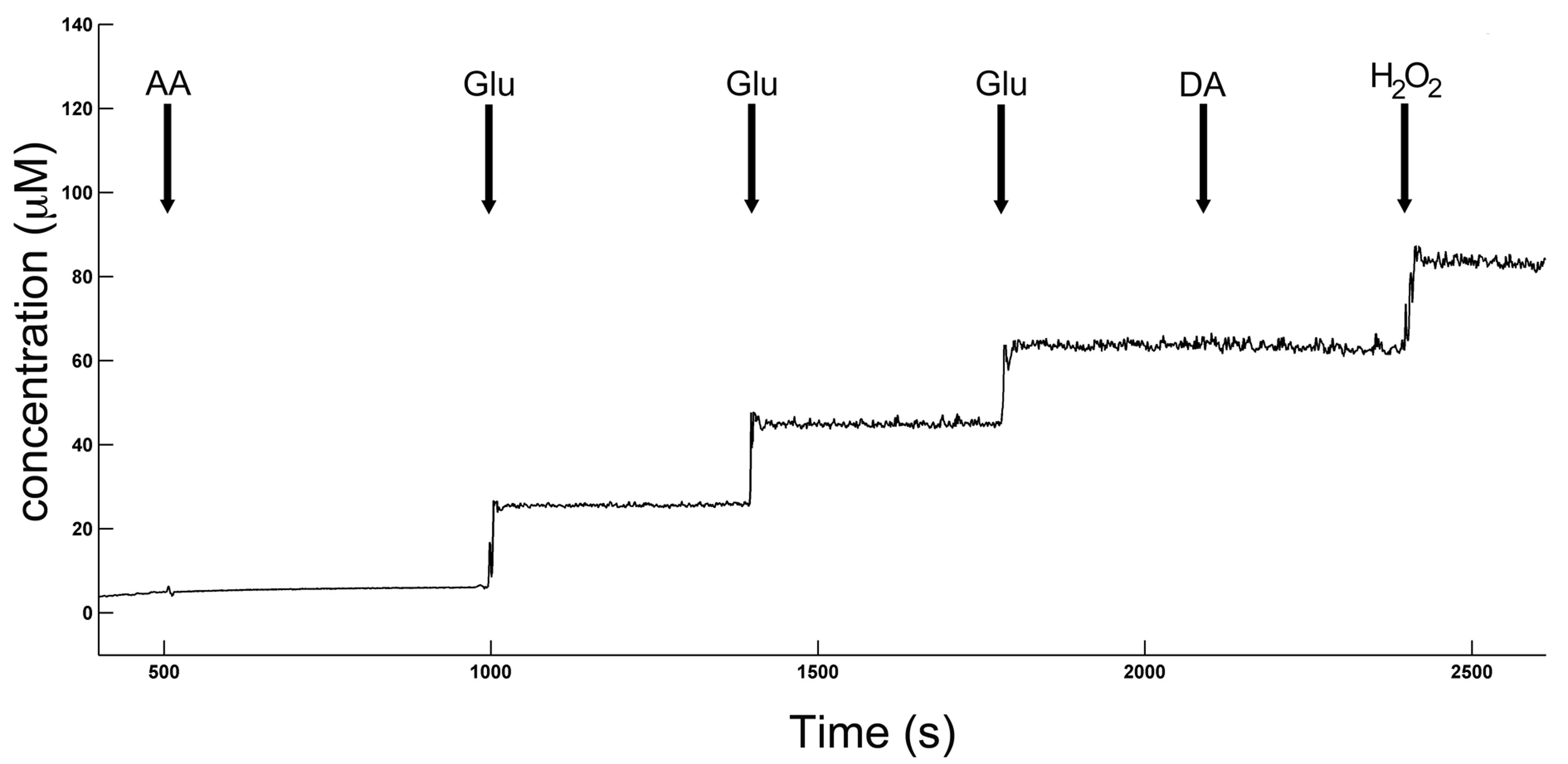

Supplement: Figure S1 — Calibration of microelectrode arrays. The electrodes were coated with glutamate oxidase and 1,3-phenylenediamine, resulting in selective recordings of glutamate (Glu, 20 µM per addition) by blocking larger molecules, such as ascorbic acid (AA, 250 µM) and dopamine (DA, 2 µm) from the electrode surface. The reporter molecule for the degradation of glutamate by glutamate oxidase, hydrogen peroxide (H2O2, 8.8 µM), was added to the calibration beaker to assure detection by the electrode. (TIF) [file pone.0055706.s001.tif]
